# Imaging diagnosis of cryptogenic multifocal ulcerous stenosing enteritis

**DOI:** 10.1186/s13244-025-01931-9

**Published:** 2025-03-07

**Authors:** Xiaoyan Zhang, Li Ma, Mengsu Xiao, Jing Qin, Mengyuan Zhou, Hong Yang, Wei Liu, Lin Cong, Weixun Zhou, Gechong Ruan, Jingjuan Liu, Guannan Zhang, Wenbo Li, Qingli Zhu

**Affiliations:** 1 Department of Ultrasound, State Key Laboratory of Complex Severe and Rare Diseases, Peking Union Medical College Hospital, Chinese Academy of Medical Sciences and Peking Union Medical College, Beijing, China; 2 Department of Gastroenterology, State Key Laboratory of Complex Severe and Rare Diseases, Peking Union Medical College Hospital, Chinese Academy of Medical Sciences and Peking Union Medical College, Beijing, China; 3 Department of Radiology, State Key Laboratory of Complex Severe and Rare Diseases, Peking Union Medical College Hospital, Chinese Academy of Medical Sciences and Peking Union Medical College, Beijing, China; 4 Department of General Surgery, State Key Laboratory of Complex Severe and Rare Diseases, Peking Union Medical College Hospital, Chinese Academy of Medical Sciences and Peking Union Medical College, Beijing, China; 5 Department of Pathology, State Key Laboratory of Complex Severe and Rare Diseases, Peking Union Medical College Hospital, Chinese Academy of Medical Sciences and Peking Union Medical College, Beijing, China

**Keywords:** Computed tomography enterography (CTE), Cryptogenic multifocal ulcerous stenosing enteritis (CMUSE), Intestinal ultrasound (IUS)

## Abstract

**Objective:**

This study aimed to summarize the intestinal ultrasound (IUS) and computed tomography enterography (CTE) features of cryptogenic multifocal ulcerous stenosing enteritis (CMUSE) and compare the performance of IUS and CTE in the evaluation of CMUSE in a single tertiary center.

**Methods:**

Clinically or pathologically confirmed CMUSE patients between December 2009 and April 2023 were recruited. Imaging features of CMUSE patients who underwent both IUS and CTE were summarized retrospectively.

**Results:**

Twenty-nine patients were included. All patients were found to have ileum involvement, with the majority (96.6%, 28/29) showing superficial ulcers and stenosis at endoscopy. Nineteen patients who underwent both IUS and CTE during the same period were identified for image review. Intestinal lesions were present in 19 patients (100%) both on IUS and CTE. IUS features of CMUSE included minimal to moderate thickened small bowel wall with over half of the patients presenting with hypoechogenicity and vague stratification, over one-third of patients exhibiting proximal bowel dilation and increased bowel wall vascularity in most patients; on CTE, it presented as slight to moderate thickened bowel wall with mural enhancement, multiple short circumferential strictures and mild proximal bowel dilation in most patients. There was no statistically significant difference between IUS and CTE in detecting lesions (19/19 vs. 19/19), bowel wall thickening, bowel strictures (*p* = 0.727), and bowel wall vascularity (*p* = 0.375).

**Conclusion:**

IUS features of CMUSE were comparable with CTE in detecting lesions, bowel wall thickening, strictures and bowel wall vascularity, suggesting that IUS could serve as a radiation-free imaging modality for the diagnosis and surveillance of CMUSE.

**Critical relevance statement:**

This pathology is relevant for gastroenterologists, radiologists, and the medical community, as well as for patients with small bowel disorders. Intestinal ultrasound could be of value and serve as a radiation-free imaging modality in assessing cryptogenic multifocal ulcerous stenosing enteritis (CMUSE).

**Key Points:**

- More data are needed to characterize the intestinal ultrasound (IUS) findings of cryptogenic multifocal ulcerating stenosing enteritis (CMUSE).
- IUS features of CMUSE manifested as thickened bowel wall, with more than half of the patients presenting with hypoechogenicity with vague stratification.
- Computed tomography enterography (CTE) features of CMUSE included bowel wall thickening with mural enhancement, multiple short circumferential strictures, and mild small intestine dilation.
- IUS and CTE were comparable in detecting lesions, bowel wall thickening, bowel strictures, and bowel wall vascularity.

**Graphical Abstract:**

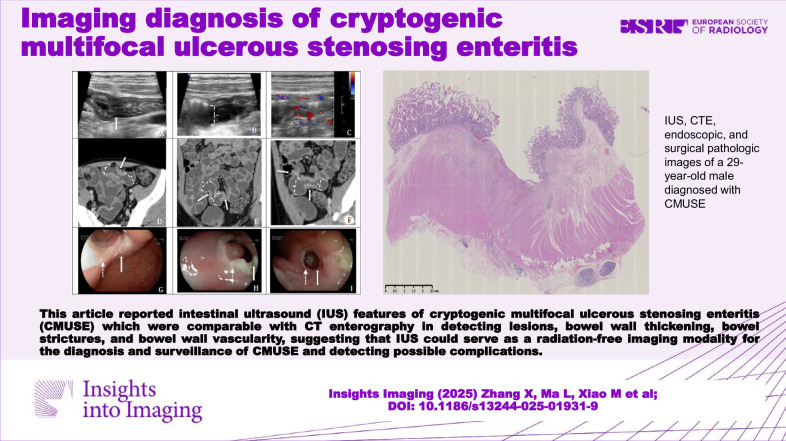

## Introduction

Cryptogenic multifocal ulcerating stenosing enteritis (CMUSE), is a rare condition that causes relapsing small bowel obstruction and bleeding due to short-segment circumferential strictures and multiple shallow ulcers of the small intestine. Clinical symptoms of CMUSE include abdominal pain, obscure intestinal bleeding and anemia, with a relapsing-remitting course despite surgery and anti-inflammatory treatment, which are non-specific [1]. CMUSE is frequently misdiagnosed as other ulcerative small bowel diseases, such as Crohn’s disease (CD) or nonsteroidal anti-inflammatory drugs (NSAID) induced enteropathy [2, 3]. Endoscopy plays an essential role in the diagnosis, management, prognosis, and surveillance of small bowel diseases, allowing for biopsy and histological evaluation [4]. However, it has limitations for the assessment of the complications [5], and the endoscope may not reach the stenosis segments, making it difficult to obtain biopsy samples. Therefore, cross-sectional imaging assistance is warranted in the diagnosis and management of CMUSE.

Computed tomography enterography (CTE) can demonstrate bowel involvement, distribution, mural enhancement patterns and extra-intestinal manifestations. The most common imaging features of CMUSE on CTE included multiple short circumferential strictures (< 2 cm in length; < 1 cm thick) with layered hyperenhancement pattern (> 90%) most commonly involving the ileum (90%) [6, 7]. Intestinal ultrasound (IUS), as an important tool for the management of small bowel disorders, has the advantage of being well-tolerated, radiation-free, repeatable, generally available and less expensive. Recently, all newly diagnosed CD patients have been recommended to undergo bowel assessment with IUS, MR enterography and/or capsule endoscopy [8]. However, there are few reports on the IUS features in patients with CMUSE, and most of them presented as case reports.

Therefore, the aim of this study was to retrospectively review and summarize the IUS and CTE imaging features of CMUSE and compare the performance of IUS and CTE in the evaluation of CMUSE patients in a single tertiary center.

## Materials and methods

### Patients

This study was approved by the Institutional Review Board of Peking Union Medical College Hospital. The requirement for informed patient consent was waived. A total of 29 patients were retrospectively enrolled in the study between December 2009 and April 2023. Two gastroenterologists with more than ten years of experience reviewed the clinical, laboratory (e.g., hemoglobin, C-reactive protein, auto-antibodies), radiological, endoscopic and treatment (e.g., medications/surgery) data. The reference diagnostic criteria were as follows: (1) refractory and occult blood loss from the gastrointestinal tract; (2) chronic or relapsing ulcerative stenosis and abdominal pain; (3) unexplained small bowel strictures; (4) superficial ulcers affecting the mucosa and submucosa; (5) no signs of systematic inflammation except during acute ileus; (6) improvement after administering corticosteroids; (7) the absence of a relatively more common etiology of small intestine ulcerative disease (e.g., CD, NSAID enteropathy, celiac disease, and small bowel malignancies) [7, 9]. CMUSE typically manifests as superficial ulcers confined to the mucosa and submucosa on surgical histopathology. The diagnosis is confirmed in patients who undergo surgery, with histopathology demonstrating these characteristic ulcerations. In cases where surgical resection is not performed, a combination of clinical, radiologic, endoscopic, and biopsy findings, along with improvement following treatment, is accepted as sufficient evidence for a diagnosis of CMUSE [3].

### IUS and CTE acquisition

IUS was performed according to the EFSUMB Recommendations and Guidelines for Gastrointestinal Ultrasound [10] by two experienced radiologists (Q. Zhu and W. Li) with over 5 years of IUS imaging experience, who have received systematic IUS training and completed over 500 IUS cases annually. Patients were examined after an overnight fast before examination. IUS was carried out using a Philips iU22 (Philips, Bothell, WA, USA) or SuperSonic Aixplorer (SuperSonic Imaging, SA, France) machine with convex (C5-2) and then, for a detailed examination, linear (L9-3) transducers. A thorough scanning of the colon was performed along the longitudinal axis from the ileocecal region to sigmoid, and mow-like scanning was performed to examine the jejunum and ileum. Images were consecutively recorded and labeled with body marker and detailed annotation. Color or Power Doppler flow parameters were optimized to maximize the sensitivity for the detection of vessels with low-velocity flow (5–7 cm/s) in the bowel wall. Persistence of color was set at “medium,” and the wall filter was adjusted to the lowest setting [10].

All patients underwent CTE using multidetector scanners after a standardized bowel preparation regime. Fasting was needed for 12 h prior to the examination. Patients ingested 1500–2000 mL of mannitol followed by 500 mL water over 60 min prior to the CTE examination for bowel extension. A single tube high-pressure injector was used to inject 90 mL of non-ionic contrast agent (Ultravist 370, 370 mg/mL, Bayer Pharma AG, Shanghai, China) through the cubital vein at a speed of 3.0 mL/s. The CT value was monitored at the level of the diaphragmatic aorta with a delay of 10 s. When the CT value > 100 Hu, the arterial phase was automatically triggered with a delay of 6 s, followed by a delay of 25 s for portal vein phase scanning. The scanning range was from the top of the diaphragm to the symphysis pubis in the supine position. All patients underwent plain scan, arterial phase, and portal vein phase scans. Multiple planar reformation (MPR) was used for image reconstruction for portal phase images.

### Image analysis

For IUS, the lesion location, bowel wall thickness, structure, vascularity, abdominal complications such as fistulas and abscesses were recorded. The lesion location was classified into jejunum and ileum. Stomach, duodenum and rectum were not evaluated as the low accuracy of IUS in detecting lesions in these areas [11]. Bowel wall thickness ≥ 4 mm (measured from the edge of the outer wall to the inner echogenic mucosa-gas acoustic surface) was considered pathological, it could be measured both in longitudinal and transverse sections [12]. In patients in whom more than one segment was involved, the maximum bowel wall thickness was used. The morphology of five layers of intestine was evaluated as “clear” and “vague.” Bowel strictures were assumed in the case of severe luminal narrowing in regions of bowel wall thickening (at least 3 mm), accompanied by destruction of wall layering and loss of peristaltic bowel movement with or without prestenotic bowel dilatation [13]. The Limberg score was used for grading bowel wall vascularity: grade 0, bowel wall thickness less than 3–4 mm without vascularization; grade I, bowel wall thickening without vascular signals; grade II, bowel wall thickening with short linear vascularity; grade III, bowel wall thickening with longer linear vascularity; and grade IV, bowel wall thickening with vascular signals extending into the surrounding mesentery [14]. IUS images were consecutively recorded and labeled, two experienced radiologists (Q. Zhu and W. Li) each with over 5 years’ experience in IUS imaging independently reviewed the images and analyzed without knowing the CTE and the enteroscopy results. Differences between the radiologists were resolved by consensus.

For CTE, imaging items of evaluation included the lesion location (jejunum and ileum), bowel wall thickness (mild (3–5 mm), moderate (> 5–9 mm), and marked (≥ 10 mm)), enhancement patterns, intestinal stenosis, proximal intestinal dilation and extra-enteric features, including mesenteric lymphadenopathy, fistula, sinus, and abdominal abscess. Luminal narrowing was defined as luminal diameter reduction of at least 50% compared with that of a normal adjacent bowel loop [15]. Stricture was defined as probable stricture without upstream dilation (< 3 cm), stricture with mild upstream dilation (3–4 cm) and stricture with moderate to severe upstream dilation (> 4 cm) [7, 15, 16]. Enlargement of mesenteric lymph nodes is defined as lymph nodes greater than 5 mm in short axis on IUS or CTE [17]. CTE images and multiplanar reconstruction images were prospectively interpreted by two radiologists each with more than 5 years of CTE experience in radiology. The two radiologists were blinded to the results of each other as well as to the results of all previous radiologic and nonradiologic investigations of the patient. Differences between the radiologists were resolved by consensus.

To compare the lesion location on surgical specimen, endoscopy and imaging, we adopted the topographic criteria and specific morphological criteria. The jejunum is identified in the left hypochondrium and the presence of well-represented valvulae conniventes. The ileal loops are detectable in the mesogastrium and the lower right quadrants and the valvulae conniventes gradually reduce in number and height.

### Statistics

The quantitative data are presented as the means and standard deviations or interquartile range. The qualitative data are presented as frequencies. The Shapiro–Wilk test was used to determine the presence of a normal distribution. The χ^2^ test with Yates’ correction and Fisher’s exact test were used to compare categorical variables. The differences between the distributions were evaluated using the chi-square McNemar test for categorical data. A value of *p* < 0.05 was considered statistically significant. Statistical analyses were performed with SPSS software version 20.0 (IBM, Armonk, NY, USA).

## Results

### Clinical information

**Fig. 1:**
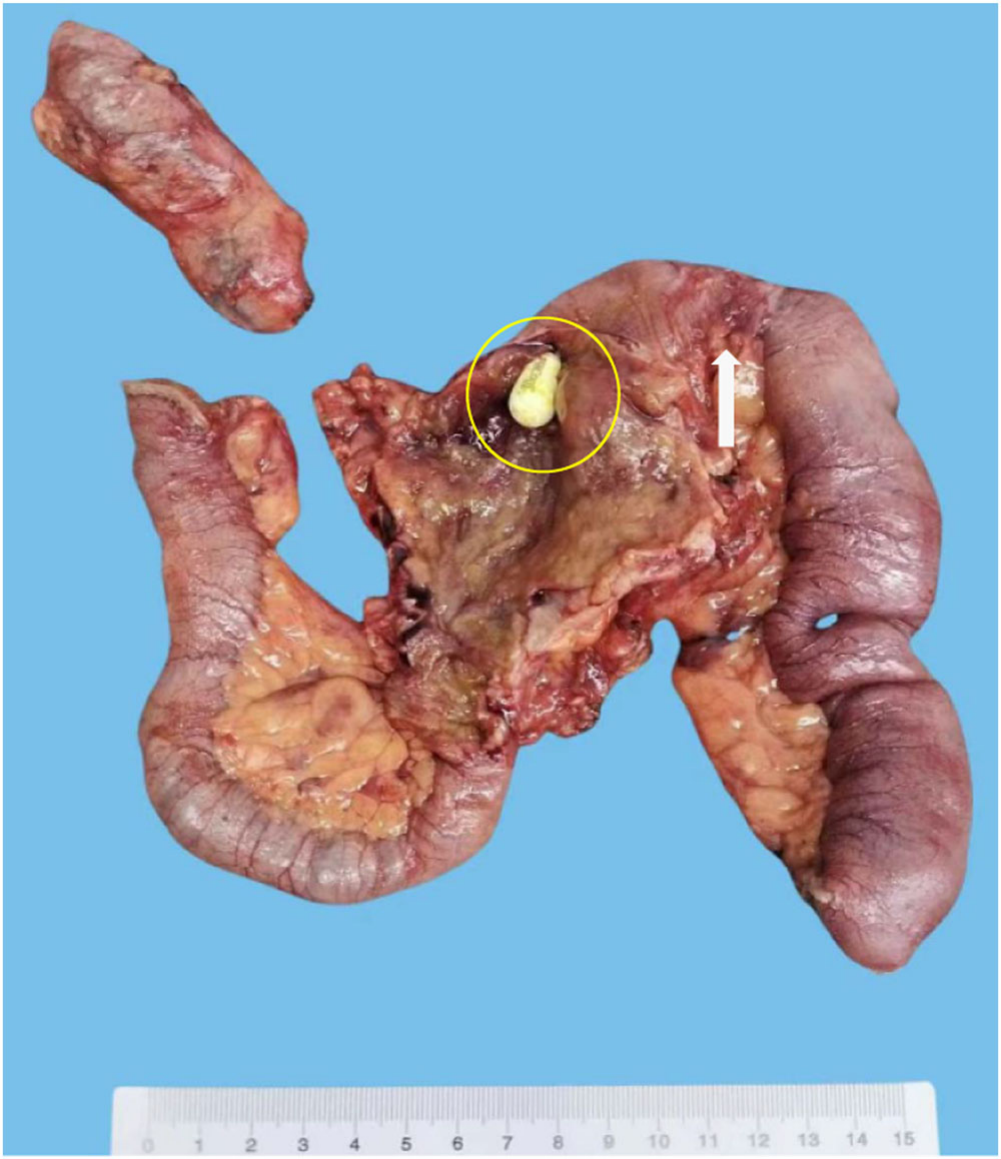
Surgical gross specimen image of a 68-year-old male patient. The patient complained of abdominal pain, incomplete intestinal obstruction and intermittent melena for 5 years, capsule endoscopy retention for 3 months. The patient underwent laparoscopic exploration and partial ileum resection due to capsule endoscopy retention. Gross specimen of resected small intestine showed short segmental stricture, angulation (arrows) with proximal small intestine dilation of 30 cm in length and 5 cm in diameter. Capsule endoscopy retention (circle) was visible at the proximal intestinal cavity of the stricture

A total of 29 patients (age: 40.3 ± 14.5 years) were included in the analysis. In our study, 13 (44.8%) patients underwent surgery and were diagnosed with CMUSE through surgical pathology, and 16 (55.2%) patients were diagnosed through a comprehensive evaluation of clinical, radiologic, endoscopic, and biopsy pathology findings. The demographic and clinical information are listed in Table 1. Anemia (23/29, 79.3%), abdominal pain (20/29, 69.0%), melena/hematochezia (15/29, 51.7%) were the main clinical manifestations. 73.3% of the patients (11/15) had a history of capsule retention (defined as the presence of the capsule in the gastrointestinal tract for ≥ 2 weeks post-ingestion [18]). Among the patients with retained capsules, five (45.5%, 5/11) patients underwent bowel resection due to capsule retention (Fig. 1), five (45.5%, 5/11) had capsule excretion after medical treatment and one (9.0%, 1/11) patient’s retained capsule was removed by transoral enteroscopy. 44.8% (13/29) of patients underwent partial small bowel resection due to intestinal obstruction, capsule retention, and intestinal bleeding. 31.0% (9/29) of the patients underwent appendectomy due to appendicitis or recurrent right lower quadrant  pain.

**Table 1:** Demographic and clinical data of CMUSE patients

Characteristics, % (*n*/*n*)	CMUSE patients
Number of patients	29
Male	58.6% (17/29)
Female	41.4% (12/29)
Age at diagnosis (years)^a^	39 (19–69)
Duration of symptoms prior to diagnosis (years)^a^	10 (0.5–25)
Symptom	
Anemia	79.3% (23/29)
Capsule retention	73.3% (11/15)
Abdominal pain	69.0% (20/29)
Melena	34.5% (10/29)
Weakness	20.7% (6/29)
Hematochezia	17.2% (5/29)
Prior obstructions	17.2% (5/29)
Diarrhea	17.2% (5/29)
Extra-intestinal manifestations	
Laboratory findings at diagnosis	
Hemoglobin (g/L)^a^	79.5 ± 32.2
Albumin (g/L)^a^	35.7 ± 8.2
ESR (mm/h)^b^	7 (1–58)
C-reactive protein (mg/L)^b^	1.8 (0.1–98)
Positive ANA	19.0% (4/21)
Positive ASCA or ANCA	11.8% (2/17)
Treatment	
Surgery	44.8% (13/29)
Corticosteroids	41.4% (12/29)
Immunomodulator (MTX or AZA or Thd)	27.6% (8/29)
Mesalazine	27.6% (8/29)

*ESR* erythrocyte sedimentation rate, *ANA* anti-nuclear antibody, *ANCA* anti-neutrophil cytoplasmic antibody, *ASCA* anti-Saccharomyces cerevisiae antibodies, *MTX* methotrexate, *AZA* azathioprine, *Thd* thalidomide

^a^ Means ± standard deviations

^b^ Median (min–max)

### Laboratory findings

Most CMUSE patients (79.3%, 23/29) developed anemia. The mean hemoglobin level of patients with CMUSE was 79.5 ± 32.2 (range 40–166) g/L. 17.2% (5/29) of the patients with CMUSE were observed to have elevated ESR levels (7 mm/h, range 1–58 mm/h). High sensitivity C-reactive protein (hsCRP) was elevated in 34.5% (10/29) of the CMUSE patients. 37.9% (11/29) of patients had albumin levels lower than the normal reference values (35.7 ± 8.2 g/L, range 10–50 g/L). Anti-nuclear antibodies (ANA) were positive in 19% (4/21) of the patients, and anti-Saccharomyces cerevisiae antibodies (ASCA) or anti-neutrophil cytoplasmic antibodies (ANCA) were positive in 11.8% (2/17) of the patients.

### Endoscopic features

**Fig. 2:**
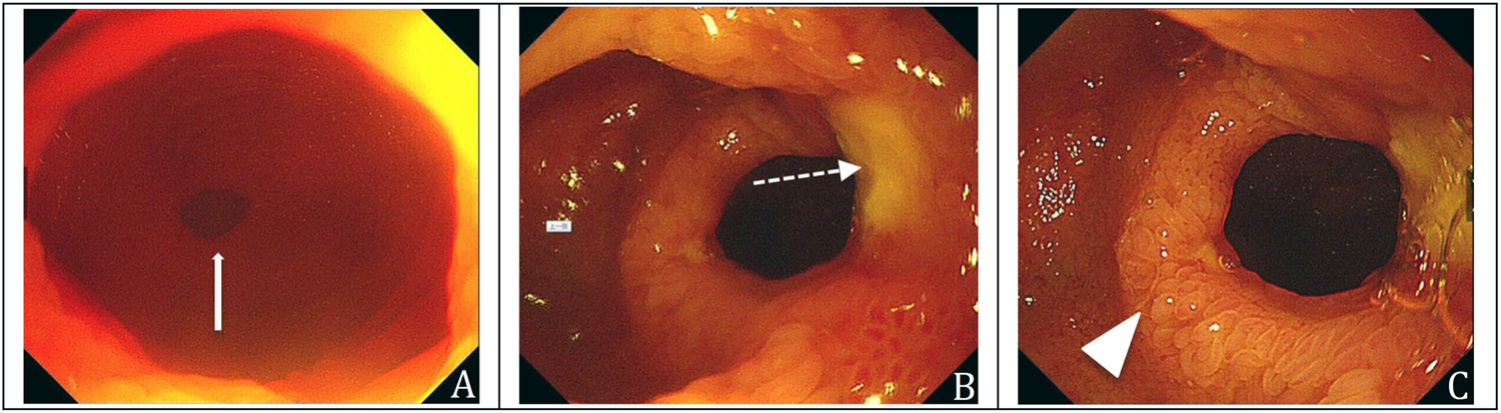
Endoscopic images of a 31-year-old male diagnosed with CMUSE for 4 years. The patient was admitted due to capsule endoscopy retention. **A**, **B** The transanal enteroscopy showed centripetal annular stricture (solid white arrows) with ulcer (dotted white arrow) in the ileum. **C** Intestinal mucosal congestion and edema with villous swelling were observed (triangle)

Jejunum was defined as the small intestine that spans from 20 cm to 200 cm of the small intestine by oral route. Ileum was defined as the small intestine that spans from 200 cm of the small intestine to ileocecal valve by oral and from ileocecal valve to 130 cm of the small intestine by anal route [19]. Endoscopically, ileum was involved in every patient (100%, 29/29). Among them, 6 (20.7%, 6/29) cases were simultaneously involved in jejunum. Most patients (96.6%, 28/29) manifested as small intestinal ulcers and strictures (Fig. 2), with 53.6% (15/28) presenting with circumferential ulcers, one patient manifested as intestinal strictures without ulcer. All ulcers observed in CMUSE patients were superficial limited within mucosal and submucosal layers. 44.8% (13/29) of the patients underwent partial bowel resection and multiple (median 3, range 2–7) superficial ulcers and strictures were found in surgery pathology, with ulcers located on strictures. 55.2% (16/29) of the patients treated with medication manifested as multiple (median 2, range 2–7) superficial ulcers and strictures at endoscopy, with ulcers located on strictures.

### Imaging characteristics of CMUSE

Nineteen patients who underwent both IUS and CTE during the same period were identified for image review. Clinical, radiologic and endoscopic features in 19 patients are summarized in Table 2.

**Table 2:** Clinical, radiologic and endoscopic information in 19 CMUSE patients

Patient no. /sex/age (y)	Symptom	Duration (y)	Hb (g/dL)	Disease location	Gross pathologic findings			CT findings		IUS findings		Endoscopy	
					No. of strictures	Ulcers	Resected bowel length (cm)	No. of strictures	Bowel dilatation	No. of strictures	Bowel dilatation	Capsule endoscopy	Enteroscopy
1/M/69	Hematochezia	4	60	Ileum	NA	NA	NA	Multiple	Present	Multiple	Present	Not done	Multiple ulcers
2/M/59	Abdominal pain	17	85	Ileum	NA	NA	NA	Multiple	Present	Multiple	Present	Not done	Multiple ulcers and strictures
3/M/68	Abdominal pain, subileus	5	142	Ileum	2	Present	20 cm	Multiple	Present	Multiple	—	Multiple ulcers and strictures, Retained	Strictures, angulation
4/M/39	Abdominal pain, subileus	4	60	Ileum	NA	NA	NA	Multiple	Present	Multiple	Present	Not done	Multiple ulcers and strictures
5/F/42	Abdominal pain, anemia	12	53	Ileum	5–6	Present	50 cm	Multiple	Present	Multiple	—	Multiple ulcers, Retained	Multiple ulcers and strictures
6/F/34	Abdominal pain, diarrhea	10	85	Ileum	NA	NA	NA	Multiple	Present	Multiple	Present	Multiple ulcers and strictures, Retained	Multiple ulcers and strictures
7/M/19	Abdominal pain, melena	10	80	Ileum, jejunum	6	Present	50 cm	Multiple	Present	Multiple	Present	Not done	Multiple ulcers and strictures
8/M/63	Abdominal pain	10	109	Ileum	NA	NA	NA	Multiple	Present	Multiple	—	Multiple ulcers and strictures	Multiple ulcers and strictures
9/F/50	Hematochezia, anemia	5	80	Ileum	NA	NA	NA	Multiple	—	Multiple		Multiple ulcers and strictures	Multiple ulcers and strictures
10/F/32	Abdominal pain, melena	25	60	Ileum	7	Present	100	Multiple	Present	Multiple	Present	Multiple ulcers and strictures, Retained	Multiple ulcers and strictures
11/M/36	Melena	1	46	Ileum	NA	NA	NA	1	Present	1	—	Multiple ulcers and strictures	Multiple Ulcers
12/M/30	Abdominal pain	2	166	ileum	NA	NA	NA	Multiple	—	Multiple	—	Multiple ulcers and strictures, Retained	Multiple ulcers and strictures
13/M/61	Hematochezia	13	64	ileum	NA	NA	NA	Multiple	Present	Multiple	—	Multiple ulcers and strictures, Retained	Multiple ulcers and strictures
14/M/32	Abdominal pain	2	86	Ileum	NA	NA	NA	Multiple	—	Multiple	—	Not done	Multiple ulcers and strictures
15/F/41	Abdominal pain, melena	2	40	Ileum	NA	NA	NA	Multiple	—	Multiple	—	Ulcer and stricture	Multiple ulcers and strictures
16/F/61	Abdominal pain, subileus	20	65	Ileum, jejunum	3	Present	140 cm	Multiple	—	Multiple	—	Not done	Congestive edema
17/M/29	Abdominal pain, melena	15	70	Ileum	7	Present	70 cm	Multiple	Present	Multiple	—	Multiple ulcers and strictures	Multiple ulcers and strictures
18/F/39	Abdominal pain, vomiting	4	89	Ileum	3	Present	15 cm	Multiple	—	Multiple	Present	Not done	Multiple ulcers and strictures
19/M/27	Abdominal pain, ileus	2	65	Ileum	NA	NA	NA	Multiple	Present	Multiple	—	Not done	Multiple ulcers and strictures

—: Absent; Multiple: ≥ 2

**Fig. 3:**
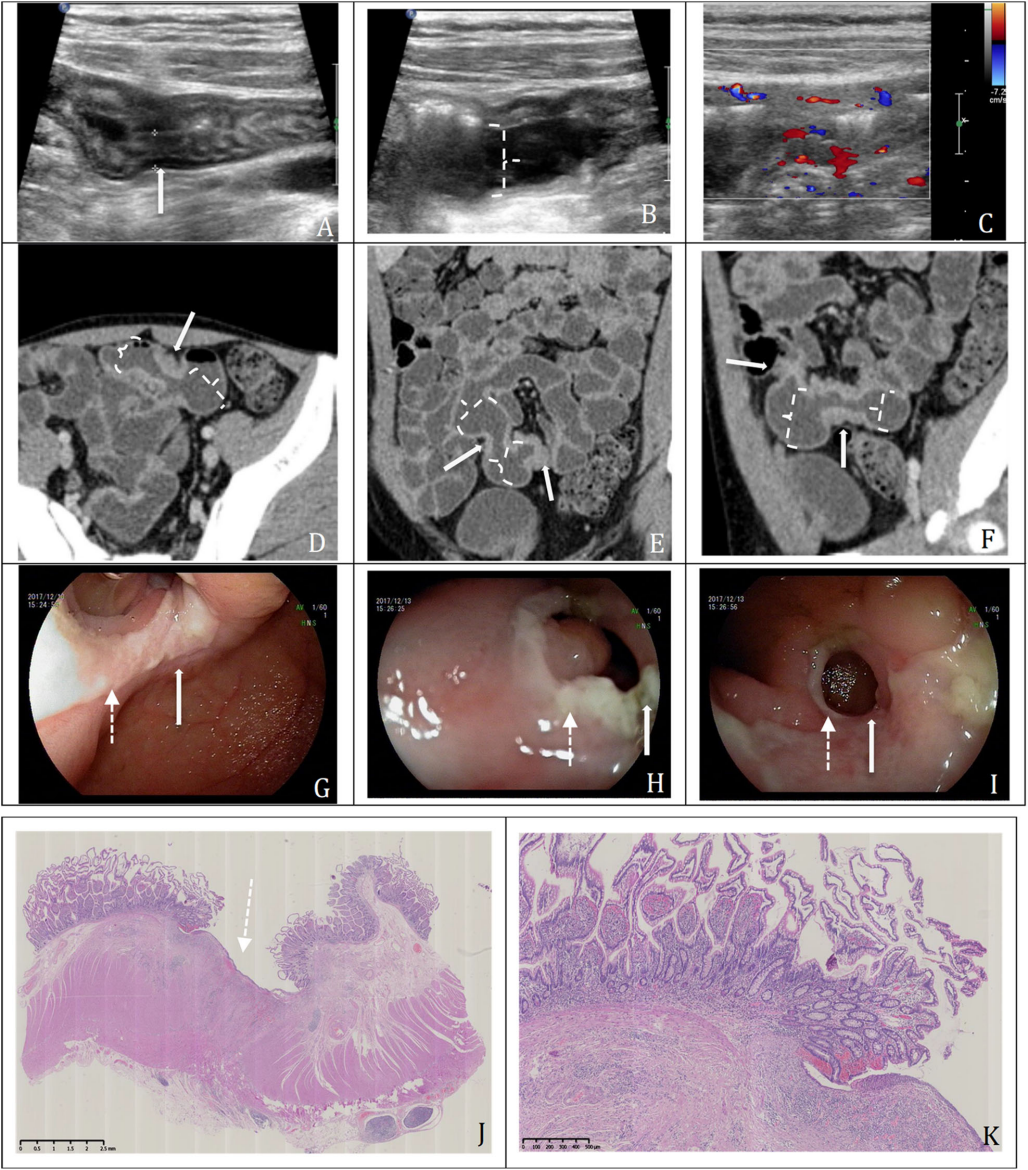
IUS, CTE, endoscopic and surgical pathologic images of a 29-year-old male diagnosed with CMUSE. The patient was admitted due to melena and intermittent abdominal pain for 15 years. The blood test showed low hemoglobin count as low as 70 g/L. **A**, **B** Longitudinal sonogram of the involved intestine showing bowel wall thickening (0.6 cm) with clear stratification, segmental stricture of the lumen (solid white arrows) and mild dilation of proximal small intestine (dashed bracket); **C** color Doppler ultrasound showed longer linear vascularity on the intestinal wall (Limberg score: IV); **D** axial plane showed ileum involvement with CMUSE which demonstrated bowel wall thickening with mural enhancement, lumen narrowing (solid white arrows) and proximal intestinal dilatation (dashed bracket); **E**, **F** reconstructed coronal and sagittal planes showed two segmental ileum involvements with thickened bowel wall and mural enhancement (solid white arrows) and proximal intestinal dilatation (dashed bracket). **G**–**I** The transoral enteroscopy showed three centripetal circumferential strictures (solid white arrows) with shallow annular ulcers (dotted white arrow) in the ileum within a local range of 10–15 cm. **G**, **H**, and **I** show the first, second, and third stricture, respectively. **J** On the H&E staining of surgical specimen, there was a shallow ulcer (dotted white arrow) confined to mucosa and submucosa (magnification × 10). **K** Medium magnification showed no dysplasia and other obvious abnormalities in the mucosa around the lesion (magnification × 50)

**Fig. 4:**
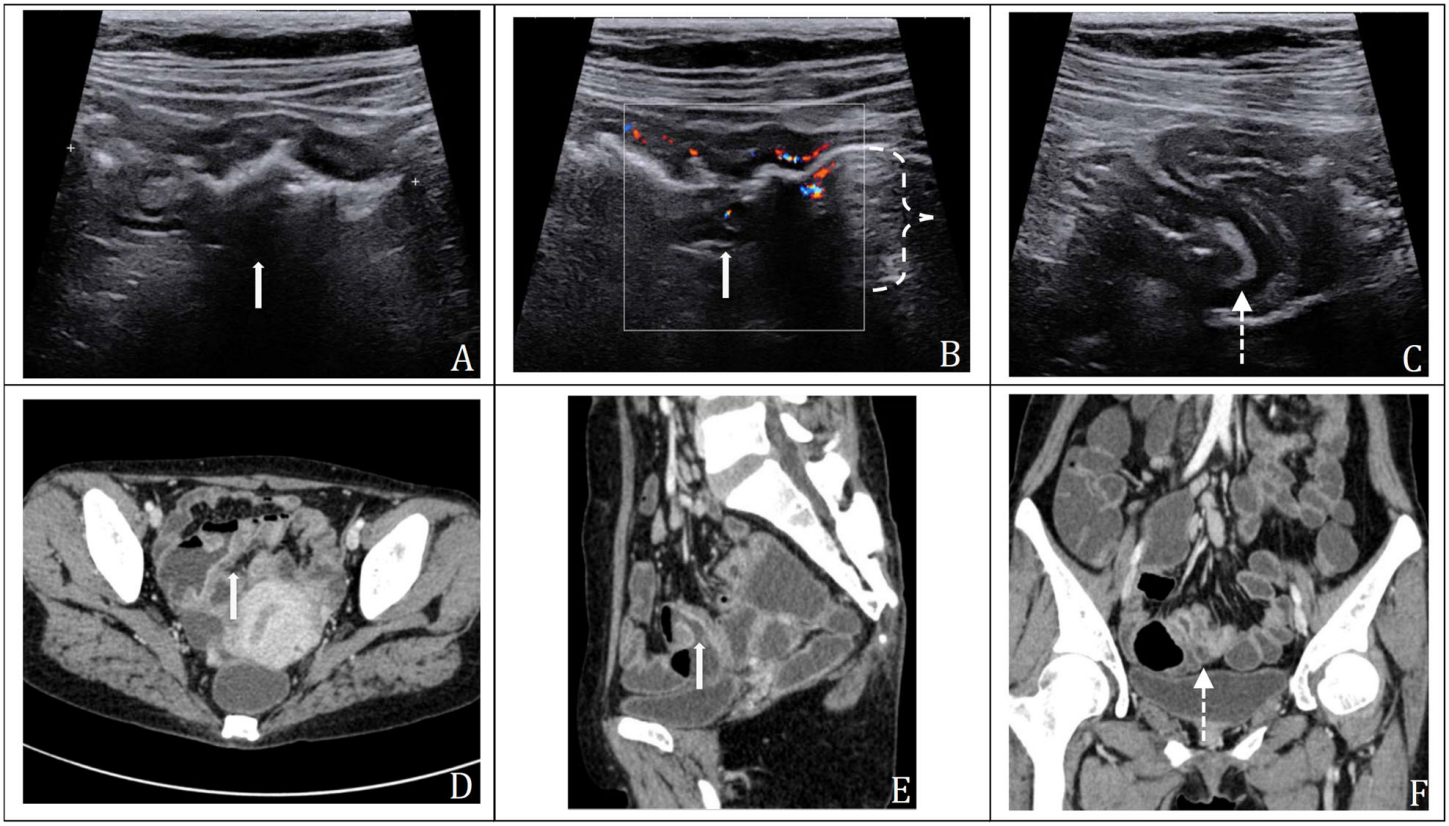
IUS and CTE images of a 41-year-old female diagnosed with CMUSE. The patient was admitted due to melena and intermittent abdominal pain for 2 years. The blood test showed a low hemoglobin count as low as 40 g/L. **A**, **B** Longitudinal sonogram of the involved intestine showed a short segmental lesion, bowel wall thickening (0.6 cm) with clear stratification (arrows); **B** color Doppler ultrasound showed longer stretches vascularity on the bowel wall (Limberg score: III). The proximal small bowel dilation (2.0 cm in diameter, dashed bracket) suggesting the stricture (arrow); **C** another segmental twisted small intestine with thickened bowel wall (dot-dashed white arrow) in the pelvic cavity was visible; **D**, **E** axial plane and reconstructed sagittal plane showing small bowel wall thickening with mural enhancement (arrows), lumen narrowing and proximal intestinal dilation; **F** reconstructed coronal plane showing another segmental twisted small intestine with thickened bowel wall and mural enhancement (dot-dashed white arrow). CTE confirmed the lesions displayed by IUS

On IUS, intestinal lesions were present in 19 patients (100%, 19/19) (Table 3). All patients had multifocal (≥ 2) bowel wall thickening. Ileum was the most common site of involvement and found in every patient (100%, 19/19), 2 (10.5%, 2/19) of these patients had concomitant jejunal lesions. Isolated disease in the jejunum did not occur. The small intestine wall was slightly to moderately thickened (median 5 mm, range 4–8 mm), and more than half of the patients presented hypoechoic bowel wall with vague stratification (11/19, 57.9%). Intestinal lesions were classified into grade I to grade IV with Limberg score [grade I, 26.3% (5/19); grade II, 36.8% (7/19); grade III, 5.3% (1/19); grade IV, 31.6% (6/19)]. Bowel strictures were identified in 11(57.9%, 11/19) patients. Proximal intestinal dilation was seen in 7 (36.8%, 7/19) patients. The upstream small bowel luminal diameter ranged from 2 to 3.5 cm (median 2.7 cm). In terms of extra-intestinal manifestations, enlarged mesenteric lymph nodes were present in six patients (31.6%, 6/19), and ascites in three patients (15.8%, 3/19). No fistulas or abscesses were found. Figure 3A–C presents ultrasonic images of a male CMUSE patient, showing multifocal bowel wall thickening with long branches of vascularity, lumen narrowing and proximal intestinal dilation. Figure 4A–C presents ultrasonic images of a female CMUSE patient, showing multifocal bowel wall thickening (0.6 cm) with clear stratification and longer stretches vascularity on the bowel wall (Limberg score: III).

**Table 3:** IUS and CTE features of the CMUSE patients

Intestinal lesions	IUS % (*n*/*n*)	CTE % (*n*/*n*)
Location		
Ileum only	89.5 (17/19)	89.5 (17/19)
Ileum and jejunum	10.5 (2/19)	10.5 (2/19)
Bowel wall stratification		
Clear	42.1 (8/19)	NA
Vague	57.9 (11/19)	NA
Limberg score/		
I	26.3 (5/19)	NA
II,III and IV	73.7 (14/19)	NA
Max bowel wall thickness (mm), median (range)	5 (4–8)	5 (3–8)
Stricture and small bowel dilation	57.9 (11/19)	68.4 (13/19)
Diameter of prox bowel dilation (cm), median (range)	2.7 (1.5–3.5)	3.0 (2.0–3.9)
Ascites	15.8 (3/19)	None
Mesenteric lymph nodes	31.6 (6/19)	68.4 (13/19)

*Prox* proximal, *NA* not applicable, *IUS* intestinal ultrasound, *CTE* computed tomography enterography, *CMUSE* imaging diagnosis of cryptogenic multifocal ulcerous stenosing enteritis

On CTE, intestinal lesions were present in 19 (100%, 19/19) patients (Table 3). All patients (100%, 19/19) had multifocal (≥ 2) bowel wall thickening (median 5 mm, range 3–8 mm). Ileum was also the most involved location (19/19, 100%), 2 (10.5%, 2/19) of these patients had concomitant jejunal lesions. Isolated disease in the jejunum did not occur. Most patients (89.5%, 17/19) had mild to moderate bowel wall thickening and mural enhancement, another two patients (10.5%, 2/19) showed suspicious slight bowel wall thickening without mural enhancement on CTE. Short-segment circumferential strictures of the small intestine and mild small intestine dilation (median 3.0 cm, range 2.0–3.9 cm) were found in 68.4% of patients (13/19) on CTE. Enlarged mesenteric lymph nodes were present in 68.4% (13/19) of patients. No fistulas or abscesses were found. Figure 3D–F presents CTE images of a male CMUSE patient showing multifocal bowel wall thickening with mural enhancement, narrowing of the lumen and proximal intestinal dilation. The transoral enteroscopy showed three centripetal circumferential strictures with shallow annular ulcers in the ileum within a local range of 10–15 cm (Fig. 3G–I). The H&E staining of surgical specimen showed a superficial ulcer and no obvious abnormalities in the mucosa around the lesion (Fig. 3J, K). Figure 4D–F presents CTE images of a female CMUSE patient, showing multifocal bowel wall thickening with mural enhancement, lumen narrowing and proximal intestinal dilation. The transanal enteroscopy confirmed two circumferential strictures in the ileum located 150 cm away from the ileocecal valve, with local shallow circular ulcers and some fresh bleeding.

There was no statistically significant difference between IUS and CTE in detecting lesions (19/19 vs. 19/19), bowel wall thickening (mild to moderate), bowel strictures (*p* = 0.727), and bowel wall vascularity (*p* = 0.375) (Table 4). Endoscopy could observe more strictures (19/19 vs. 11/19 vs. 13/19, χ^2^ = 11.366, *p* = 0.004) than IUS and CTE and could clearly observe the number, size, morphology and depth of ulcers, while IUS and CTE could not observe ulcers, especially shallow ulcers.

**Table 4:** Comparison of diagnostic results between IUS and CTE in CMUSE patients (*n* = 19)

IUS							
		Bowel wall thickening		Bowel stricture		Bowel wall vascularity	
		+	−	+	−	+	−
CTE							
	+	19	0	8	5	13	4
	−	0	0	3	3	1	1
*p*-value		NA		0.727		0.375	

*IUS* intestinal ultrasound, *CTE* computed tomography enterography, *NA* not applicable, *CMUSE* imaging diagnosis of cryptogenic multifocal ulcerous stenosing enteritis

## Discussion

This is a single tertiary care center experience that described the clinical and imaging characteristics of CMUSE in a Chinese population. We found some characteristics of CMUSE: clinically, the involved population was mainly middle-aged and young adults, with severe anemia and high capsule retention rate; typical manifestation could be found on cross-sectional imaging including minimal to moderate bowel wall thickening with multiple short segmental lumen strictures, most commonly involving the ileum, and less peripheral exudation.

CMUSE has been known to occur in the small bowel. Ileum was the predominant location of CMUSE involvement in our study and previous reports [3, 6, 7, 20]. The study reported by Ramos and Hwang [6, 7] describes the clinical and imaging characteristics of CMUSE. Major features of CMUSE included multiple, short, circumferential strictures with moderate wall thickening, and hyperenhancement located in the ileum and/or jejunum. Some ulcerative lesions or wall thickening progressed into strictures on follow-up small bowel series/CT, and some strictures revealed recurrent ulceration on follow-up small bowel series [6, 7]. These features are invaluable information in the multidisciplinary diagnostic evaluation of CMUSE and the differentiation of CMUSE, CD, and NSAIDs. In our cohort, major IUS features included mild to moderate bowel wall thickening, multiple short segmental circumferential strictures, and increased bowel wall vascularity in most patients. We described equal accuracy of IUS compared with CTE for the evaluation of disease. Given the absence of ionizing radiation and its noninvasive and convenient performance, IUS is expected to play an important role in the diagnosis and follow-up of CMUSE patients. In this retrospective study, we did not compare the number of bowel strictures among IUS and CTE [6, 7]. It is difficult for IUS to detect mild strictures without taking oral contrast solution. IUS may not be as good as CTE/MRE in terms of exact lesion numbers. More studies are needed to verify the value of IUS for the evaluation of CMUSE.

Capsule retention occurs in approximately 2–13% of patients with small bowel disorders and is most likely due to small bowel strictures [21, 22]. Our study showed that CMUSE patients had a high capsule retention rate of up to 73.3%. Research has shown that CTE evaluation or patency capsules before undergoing capsule endoscopy could decrease the capsule retention rate [21, 23, 24]. Since there was no statistically significant difference between IUS and CTE in detecting bowel strictures, thus IUS could be served as a radiation-free imaging modality in combination with patency capsule to predict gastrointestinal tract patency and decrease capsule retention rate. One limitation of IUS and CTE is the inability to display small bowel mucosal abnormalities like ulcers, especially aphthous ulcer [25, 26].

Histopathology of CMUSE reveals an atypical mixed inflammatory infiltrate composed of plasma cells, monocytes, neutrophils and eosinophils and shallow ulcers usually restricted to the mucosa and the submucosa [9, 27]. Diagnosis of CMUSE is based on history, clinical, radiologic, enteroscopic, and histology features. In differential diagnosis of CMUSE, first of all, CD must be excluded. CD often show asymmetric mural abnormalities involving long segments of small bowel, with mesenteric dominancy, mesenteric shortening with pseudosacculation, penetrating complications, and associated mesenteric findings on radiology [16]. Disease location (e.g., colon, duodenum, stomach, and esophagus), ulcer locations, ulcer depth, and transmural inflammation and extra-enteric complications (e.g., primary sclerosing cholangitis, sacroiliitis, etc.) can also be used as differentiating signs for CD. The different imaging features may be attributed to the relatively shallow ulcerations confined to the mucosa and submucosa in CMUSE, which is characterized by inflammatory infiltration of neutrophils and eosinophils [9]. The short circumferential ulcers without progression to fistula, fissures or cobblestone appearance are specific at enteroscopy for CMUSE [28]. Overlapping imaging features that are less specific include the degree of bowel wall thickening (6–9 mm), increased stratified enhancement pattern, degree of proximal bowel dilation, number and size of the mesenteric lymph nodes and the presence of mesenteric fluid.

The imaging features of NSAID enteropathy are also similar to CMUSE. 90% of NSAID enteropathy strictures were circumferential, ring-like, short in length, and symmetric with respect to the bowel lumen. Mild to moderate segmental bowel wall thickening (5–8 mm) was observed in two-thirds of patients, and half demonstrated mild to moderate mural hyperenhancement and mild proximal bowel dilation (3.8 cm) [29]. Consequently, differentiating the two entities on imaging features alone is insufficient, thus clinical (NSAID use), pathology, and radiology data need to be integrated for accurate diagnosis. NSAID enteropathy has a long history of medication and can be recovered after withdrawal [30]. Chronic enteropathy associated with SLCO2A1 gene (CEAS) is a newly recognized, rare, chronic enteropathy characterized by multiple ulcerations and strictures in the small bowel [31, 32]. CEAS has clinical and imaging characteristics similar to those of CMUSE, except for the female predominance of CEAS [1, 6, 7]. Genetic testing will help to distinguish between CEAS and CMUSE. Other small intestinal diseases must be excluded, too, such as intestinal tuberculosis and other chronic infections of the small bowel and malignancies such as small intestinal lymphoma.

Our study has some limitations. First, although the sample size was relatively large, it was still a small sample size study and may have some selection bias. Second, due to the rarity of CMUSE, this study is a retrospective study with enrolled patients between December 2009 and April 2023, and CTE was a routine imaging modality in our hospital, while MRE was less commonly used in clinical practice during this period, only one patient underwent MRE examination, therefore MRE was not included in the study and compared to IUS. Comparative study of IUS and MRE is expected to be conducted in the future. Third, the imaging follow-up of intestinal diseases is very important, it could detect disease progression and provide important clues for diagnosis, thus the imaging follow-up of intestinal diseases will be conducted in future research. Lastly, one limitation of IUS could be the lower inter-reader agreement, consensus between readers may resolve this issue.

## Conclusion

We reported IUS imaging features of CMUSE which was comparable with CTE in detecting lesions, bowel wall thickening, bowel strictures, and bowel wall vascularity, indicating that IUS could serve as a radiation-free imaging modality for CMUSE diagnosis and follow-up, and detecting possible complications.

## Data Availability

The data underlying this article will be shared on reasonable request to the corresponding author.

## Abbreviations

ANA: Anti-nuclear antibody
ANCA: Anti-neutrophil cytoplasmic antibody
ASCA: Anti-Saccharomyces cerevisiae antibodies
CD: Crohn’s disease
CMUSE: Cryptogenic multifocal ulcerous stenosing enteritis
CTE: Computed tomography enterography
ESR: Erythrocyte sedimentation rate
IUS: Intestinal ultrasound
